# Membrane Mimetics Control Internal Hydration and Catalytic Activity of the Copper ATPase LpCopA from *Legionella pneumophila*

**DOI:** 10.3390/ijms27198870

**Published:** 2026-10-05

**Authors:** Lisa Nucke, Yun-Hsuan Huang, Satoru Tsushima, Jana Oertel, Karim Fahmy

**Affiliations:** Institute of Resource Ecology, Helmholtz-Zentrum Dresden-Rossendorf, 01328 Dresden, Germany

**Keywords:** membrane proteins, P-type ATPases, internal hydration, membrane mimetics, nanodiscs, DIBMALPs

## Abstract

Membrane protein function is strongly influenced by the surrounding lipid bilayer through specific lipid–protein interactions and collective membrane properties. Here, we investigated how the membrane environment modulates internal hydration and catalytic activity of the P_1B_-type copper ATPase LpCopA. LpCopA variants labeled with 6-bromoacetyl-2-dimethylaminonaphthalene (BADAN) were reconstituted in *Escherichia coli* lipid-doped mixed micelles, membrane scaffold protein nanodiscs, and diisobutylene-maleic acid lipid particles (DIBMALPs). Local hydration near the conserved metal-binding cysteines C382 and C384 was assessed by fluorescence spectroscopy in the presence of polyethylene glycol 1500 (PEG-1500), and ATPase activity was determined in the corresponding membrane mimetics. C382 exhibited a more hydrated local environment than C384. In mixed micelles, the osmolyte-induced spectral response corresponded to an apparent hydration volume change of approximately 800 Å^3^, equivalent to about 25 water molecules; the corresponding change in nanodiscs was less than half as large. ATPase activity increased with osmotically responsive internal hydration, whereas DIBMALPs showed minimal hydration changes and strongly reduced ATPase activity. These findings suggest that membrane-dependent transmembrane packing modulates internal hydration and LpCopA activity. Energetic estimates further support a model in which hydration-dependent transmembrane expansion against membrane lateral pressure contributes to the free-energy barrier associated with conformational transitions during the catalytic cycle.

## 1. Introduction

The flow of information and matter across biological membranes is vital for all cellular life. Membrane proteins acting as receptors of external signals or as transporters of ions and substrates fulfill these functions as crucial constituents of the lipid bilayers that make up the membranes of cells and organelles. The structure and function of membrane proteins are modulated by multiple lipid–protein interactions that minimize the free energy of the surrounding protein–lipid ensemble, for example, through hydrophobic matching of transmembrane protein domains to the lipid bilayer thickness, or by adaptation to bilayer curvature, lateral pressure or lipid asymmetry [1,2,3,4]. The physico-chemical linkage between membrane proteins and membrane lipids provides the ground on which the co-evolution of both can be retraced [5].

Although the lack of lipid–protein interactions may lead to loss of function, membrane protein function itself entails structural transitions between distinct conformations which cannot share the same energy-minimized lipid–protein interactions. This renders the lipidic phase an active component which affects the energetics of membrane protein function. Ion-transporting ATPases exhibit particularly large structural changes originally demonstrated for the Ca^2+^-transporting P_2_-type ATPase SERCA from the sarcoplasmic reticulum [6]. These transporters appear particularly well-suited to study fundamental aspects of the linkage between membrane protein function and dynamic interactions within a lipid bilayer.

P-type ATPases are found in all kingdoms of life, using the energy of ATP to build up and maintain ion gradients across biological membranes accompanied by transient auto-phosphorylation. In microorganisms, these transporters play a critical role in metal homeostasis and detoxification [7]. The Gram-negative pathogen *Legionella pneumophila* employs the Cu^+^-exporting P_1B_-type ATPase LpCopA in response to conditions encountered within alveolar macrophages after host infection [8,9]. LpCopA possesses an N-terminal heavy metal binding domain and two P_1B_-type-specific transmembrane helices which precede a core structure of six helices shared by all P-type ATPases. The core structure harbors a canonical binding site (CBS) for Cu^+^ including the conserved cysteine residues C382 and C384 on helix IV, as well as other residues that define the ion pathway through the membrane domain [10]. Accessibility and affinity of these residues is under the control of three cytosolic domains found in all P-type ATPases which transmit ATP-driven structural changes to the core domain [11,12].

For LpCopA, crystal structures have been obtained in various structural states [10,13]. Detailed kinetic characterization in mixed micelles further revealed two apparent ATP-binding affinities and a biphasic Cu^+^-dependent activation profile, leading to an extended Post–Albers reaction model [14]. Specific lipid effects on LpCopAs function have been analyzed thoroughly both in vitro and in silico. ATPase activity in mixed micelles (MMs) was found to be promoted predominantly by 1-palmitoyl-2-oleoyl-*sn*-glycero-3-phosphoglycerol (POPG) and cardiolipin. Coarse-grained molecular dynamics simulations in lipid bilayers have traced these interactions back to defined transmembrane segments and further down to single amino acid preferences for specific lipid species [15]. However, much less is known about how the ensemble properties of the membrane environment influence internal hydration and couple to catalytic function. MMs are typically used to grow crystals and to measure ATPase function, but their geometrical constraints differ markedly from those of a lipid bilayer, where lateral pressure is exerted on membrane proteins [16]. Consequently, intramolecular helix packing, hydration, and hydrophobic matching constraints differ in MMs and probably also in various membrane mimetics, other than liposomes, which are increasingly used for in vitro catalytic assays. How these reconstitutions affect the interdependence of global bilayer properties, membrane protein hydration and catalysis has not been studied systematically.

The overwhelming chemical potential of water at the lipid–protein interface favors hydrated conformations over dehydrated states. The alternating access model of the Post–Albers cycle of ion transport builds on the switch between conformations with either intra- or extracellular accessibility of an internal ion-binding site, the E1 and E2 state, respectively, with occluded states between the transition [17]. However, analyses of the E2 states of LpCopA suggested that ions are released from binding sites deep within the transmembrane domain via a water-lined pathway, without passing through an occluded intermediate, emphasizing the functional importance of internal hydration [10].

We have previously shown that the CBS cysteines of LpCopA are accessible to site-directed labeling from the aqueous phase without the need for partial unfolding, thereby preserving the TM architecture. This allowed us to probe the local dipolar environment near the CBS by attaching the solvatochromic fluorophore 6-bromoacetyl-2-dimethylaminonaphthalene (BADAN), which revealed substantial differences in local hydration dynamics near C382 and C384 [18]. Figure 1 shows the crystal structure of the LpCopA transmembrane domain with the approximate locations of the fluorophore when attached at either cysteine residue, together with crystallographically resolved water molecules. To enable site-specific labeling of the CBS cysteines in helix IV, the four cysteine residues in the C-terminal heavy metal-binding domain were replaced by serine. These features make LpCopA a particularly useful model system for addressing a fundamental question in membrane protein biophysics: how does the membrane environment affect internal hydration of the transmembrane domain, and how is this linked to catalytic activity?

To obtain a quantitative estimate of internal hydration, osmotic stress was applied to BADAN-labeled LpCopA mutants, which allows us to determine the number of osmotically exchangeable water molecules. This concept was originally developed for ion channels [19] and has been reviewed recently with a focus on experiments that demonstrated the functionally required water-driven expansion of the visual photoreceptor bovine rhodopsin, a prototypical class-A GPCR [20]. Such studies rely on observables that report on conformational transitions involving water uptake or release and are therefore sensitive to osmolytes. The osmotically modulated hydration processes are not required to take place at the site used for conformational monitoring as long as they are allosterically coupled to that site. In the present study, BADAN serves as a site-specific reporter of local polarity through its emission wavelength [21]. Its osmolyte-dependent spectral response can be used to quantitatively describe those hydration-dependent conformational equilibria of the entire LpCopA structure which are directly or allosterically linked to the polarity at the labeled sites. Here, we exploit these two causes of spectral modulations of BADAN to characterize both local hydration and global osmotically exchangeable water in LpCopA reconstituted in different membrane mimetics. The results show that ATPase activity correlates with internal hydration of the transmembrane domain and that this relationship is strongly modulated by the membrane environment.

## 2. Results

### 2.1. Osmolyte-Induced Changes in BADAN Fluorescence

Spectroscopic and functional assays were performed using BADAN-labeled and unlabeled LpCopA to address the influence of the membrane environment on internal hydration and catalytic activity, respectively.

Figure 2 shows the effect of 32.5% (*w*/*w*) polyethylene glycol 1500 (PEG-1500) on the emission spectra of BADAN attached at either C382 or C384 in four reconstitution systems: *n*-dodecyl-β-D-maltopyranoside (DDM) micelles, mixed micelles (MMs), membrane scaffold protein nanodiscs (NDs), and diisobutylene-maleic acid lipid particles (DIBMALPs). The emission spectra were fit with the sum of two Gaussian components to estimate the relative populations of weakly and strongly hydrated states near the two labeling sites. In all systems, addition of PEG-1500 shifted the spectra toward shorter wavelengths, reflected by an increased contribution of the Gaussian component with an emission maximum below 450 nm relative to the component above 485 nm.

In the absence of osmolyte, the emission maximum was consistently located at a longer wavelength for BADAN at C382 than for BADAN at C384. The magnitude of the PEG-1500-induced spectral response also generally followed the degree of basal hydration at the two sites, despite their separation by only a single proline residue (Table 1).

**Table 1:** Site-specific emission maximum shift, molecular volume change Δ*V* and number of exchangeable water molecules (#H_2_O), and ATPase activity of LpCopA in different membrane mimetic systems. The values for the molar volume change Δ*V*_m_ were derived from the slope of the regression lines in Figure 3 according to Equation (1) and converted to the molecular values Δ*V*. The corresponding numbers of water molecules were estimated using a water volume of 30.3 Å^3^. Importantly, these values represent global hydration-linked volume changes sensed by BADAN and are not assigned to the labeled sites. Taking into account that BADAN resides in a solvent-accessible protein crevice, the more expanded conformation may be favored by the label. Hence, the Δ*V* estimates represent a lower limit. BADAN-labeled LpCopA is catalytically inactive, whereas unlabeled wild-type LpCopA lacks the fluorescent reporter. In these cases, the listed criteria are not applicable (n.a.).

**Label Position**	**Mimetic** **System**	**Emission Maximum Shift/** **nm**	**∆*V*/nm^3^** **(Error)**	**#H_2_O** **(Error)**	**ATPase Activity/** **nmol P_i_ mg^−1^ min^−1^** **(Error)**
BADAN@C384	DDM	464 → 459	0.30 (0.05)	10.0 (1.5)	n.a.
	MM	465 → 461	0.81 (0.09)	27.3 (3.2)	n.a.
	ND	460 → 458	0.18 (0.04)	6.2 (1.5)	n.a.
BADAN@C382	DDM	478 → 461	0.60 (0.05)	19.9 (1.7)	n.a.
	MM	482 → 463	0.76 (0.09)	25.6 (3.0)	n.a.
	ND	460 → 453	0.27 (0.04)	9.2 (1.5)	n.a.
	DIBMALP	456 → 456	0.08 (0.13)	2.6 (4.2)	n.a.
No label	DDM	n.a.	n.a.	n.a.	0.69 (2.72)
	MM	n.a.	n.a.	n.a.	53.51 (4.00)
	ND	n.a.	n.a.	n.a.	37.23 (5.28)
	DIBMALP	n.a.	n.a.	n.a.	9.85 (6.40)

In MMs, PEG-1500 induced pronounced blue shifts at both reporter sites. The emission maximum shifted from 482 to 463 nm at C382 and from 465 to 461 nm at C384. Although the magnitude of the spectral shift differed between the two sites, both spectra showed a substantial redistribution toward the short-wavelength Gaussian component (Figure 2a,e).

In DDM micelles, PEG-1500 also induced blue shifts at both reporter positions, but the responses differed more strongly between the two sites. The emission maximum shifted from 478 to 461 nm at C382 and from 464 to 459 nm at C384, indicating a larger spectral response at C382 (Figure 2b,f).

Compared with DDM micelles and MMs, NDs showed smaller osmolyte-induced changes (Figure 2c,g). At C382, the emission maximum shifted from 460 to 453 nm, whereas only a minor shift from 460 to 458 nm was observed at C384.

The smallest hydration response was observed in DIBMALPs and was detectable only at the more strongly hydrated site, C382 (Figure 2d). DIBMALPs exhibited, already in the absence of osmolytes, the most blue-shifted BADAN fluorescence. Upon addition of PEG-1500, the emission maximum in DIBMALPs remained essentially unchanged at 456 nm. Only a modest narrowing of the emission band and a small redistribution of the Gaussian components were observed. Conceptually, the reconstitution of LpCopA in DIBMALPs was performed to provide the most native-like membrane mimetic in our comparative study. Neither transient detergent-solubilization nor the provision of non-native lipid mixtures is required. Also, the labeling of LpCopA occurred directly in *E. coli* membranes, proving the reactivity of BADAN with the native membrane-embedded LpCopA structure. It was thus surprising that LpCopA in DIBMALPs differed so markedly from its reconstitution in NDs.

In summary, the spectra in Figure 2 qualitatively rank the different LpCopA reconstitutions from to Figure 2a–d with respect to both decreasing local polarity at the CBS and decreasing sensitivity to osmotic pressure. This correlation is not surprising because less hydrated states are less sensitive to further dehydration by an osmolyte. However, osmotic effects concern the LpCopA structure as a whole and cannot be assigned to any site, including the labeled sites themselves. As long as the global hydration of LpCopA is sterically coupled to the polarity around the labeled sites, the latter will respond allosterically to the hydration state also at distant protein crevices. In the following, the site-specific response of BADAN emission to osmotic pressure is evaluated quantitatively to derive 1. global hydration-dependent molar volume changes and 2. the membrane lateral pressure under which these volume changes occur. Both physical properties can be obtained from free energy estimates of the weakly to strongly hydrated LpCopA conformational transition. Whereas the molar volume change will be translated into a number of osmotically exchangeable waters, the lateral pressure is a key property which distinguishes the tested membrane mimetics even in the case of harboring the identical lipid composition or native *E.coli* lipids.

### 2.2. Site-Dependent Osmotically Exchangeable Hydration Volumes

To relate the osmotically induced spectral changes to the number of water molecules exchanged with the aqueous phase, an apparent equilibrium constant, *K*, was defined as the ratio of the areas of the Gaussian components assigned to the apparent strongly and weakly hydrated states. The logarithm of *K* was determined for varying concentrations of PEG-1500 and plotted against the tabulated osmotic pressures of PEG-1500 solutions [22].

Figure 3 shows an approximately linear relationship between ln*K* and osmotic pressure for all reconstitution systems. This behavior indicates that, despite the simplifying two-state approximation, the spectral response can be described consistently by an apparent equilibrium of the following form:

(1) $${K\;=\;e}^{\frac{-(\Delta G_{0}\;+\;\pi \cdot {\Delta V}_{m})}{RT}},$$

where Δ*G*_0_ is the standard free energy change of the weakly to strongly hydrated state transition. The additional free energy contribution imposed by osmotic pressure is written as ΔΔ*G*_0_ = π*∙*Δ*V*_m_, where Δ*V*_m_ (>0) is the molar volume change associated with intra-membrane protein hydration and π corresponds to the osmotic pressure. The molar volume changes Δ*V*_m_ were derived from the slopes of the regression lines in Figure 3 according to Equation (1) and converted into molecular volume changes Δ*V* per LpCopA molecule. The corresponding numbers of exchanged water molecules were estimated using a molecular water volume of 30.3 Å^3^ (Table 1).

The data show that a total of about 25 internal water molecules is in osmotic equilibrium with the aqueous phase in MMs (Figure 3). The osmotic dehydration is equally sensed at both reporter sites, which nevertheless reside in unequal local dipolar environments as evidenced by their different emission maxima and band shapes irrespective of the presence of PEG-1500 (an equivalent evaluation of volume changes based on spectral decomposition on the wavenumber scale deviates within the error given in Table 1; see Figure S8).

This is in stark contrast to the osmotic response in DDM. Here, the dipolar environment of the label at C382 is linked to about 20 exchangeable water molecules, whereas only half of that value is sensed by the label at C384. In this case, two equilibrium constants with different Δ*V*_m_ are required to describe the osmotic effect.

LpCopA in NDs showed intermediate behavior. The osmotically coupled hydration volume sensed through both reporter sites was approximately half of that observed in MMs.

By comparing the effects of PEG-1500 between BADAN-labeled C382 and C384, the question arises as to what extent the interrogated sites are structurally independent. They may be linked to the same osmolyte-induced global hydration changes in MMs but clearly sense different hydration sites in NDs. We have addressed this question by exposing LpCopA in NDs to a smaller osmolyte PEG-200. In fact, the smaller osmolyte had virtually no effect on the BADAN emission when the label was linked to C382. In contrast, PEG-200 induced a fluorescence red-shift of the label at C384 (Figure 4).

DIBMALPs displayed a negligible amount of osmotically exchangeable water among the examined reconstitution systems.

### 2.3. Relationship Between Internal Hydration and ATPase Activity

The pronounced differences in internal hydration among the reconstitution systems prompted us to ask whether these differences correlate with ATPase activity. Figure 5 and Figure S4 compare the ATPase activity of unlabeled, wild-type LpCopA in MMs, DDM, NDs and DIBMALPs, measured as the time-dependent release of inorganic phosphate in the presence of ATP and Ag^+^ as a substitute for Cu^+^ [23]. After 30 min, the highest phosphate release was observed in MMs, followed by NDs, whereas minimal activity was detected in DDM micelles and DIBMALPs. The specific ATPase activities determined for all reconstitution systems are summarized in Table 1.

Figure 5 relates the number of osmotically exchangeable water molecules reported by BADAN@C382 to the specific ATPase activity of unlabeled wild-type LpCopA in the corresponding membrane mimetic system. With the exception of DDM micelles, which were catalytically inactive, higher ATPase activity was associated with a larger amount of osmotically exchangeable water.

## 3. Discussion

### 3.1. Osmotic Stress Reveals Membrane-Dependent Internal Hydration

The BADAN measurements demonstrate that both basal hydration and the amount of osmotically exchangeable water in LpCopA depend strongly on the membrane mimetic. C382 consistently reported a more hydrated local environment than C384, confirming that the two closely spaced residues of the CBS experience distinct dipolar environments. A blue shift of BADAN emission is associated with reduced water-dependent dipolar relaxation in the chromophore’s molecular environment, as previously observed in phospholipid membranes and synthetic transmembrane segments [21,24]. Thus, the BADAN reporters at both CBS cysteines respond to osmotic changes in hydration that are directly or allosterically coupled to their local environments.

The spectral measurements probed the basal ligand-free state of LpCopA in the absence of transported metal and nucleotide. This apo state preferentially adopts an inward-facing E1 conformation as shown for the E1 ground-state preference of apo AfCopA crystallized in the absence of any substrate [25] and the inward-open apo E1 conformation observed for a P_1B_-type ATPase, HMA4, from *Oryza sativa* [26]. Thus, an E1-like state most likely dominates the conformational ensemble of LpCopA under our experimental conditions. However, ligand-free P-type ATPases can remain conformationally dynamic, as demonstrated by single-molecule FRET measurements of *Listeria monocytogenes* Ca^2+^-ATPase (LMCA1), showing reversible sampling of E1- and E2-like states in the absence of substrates [27]. Accordingly, our data address the hydration of the TM domain of apo LpCopA within its entire accessible conformational space and were not designed to trap an intermediate of the Post–Albers cycle.

Since BADAN only reacts with natively accessible metal binding pockets, wider conformations around the CBS may have been preferentially labeled. Therefore, we regard the derived numbers of osmotically exchangeable water as lower estimates. However, the same site-specific reporter constructs were compared across the different membrane mimetics. Therefore, their relative ranking with respect to the basal hydration of LpCopA is unaffected.

MMs supported the largest osmotically exchangeable hydration volume, although pure detergent might intuitively be expected to provide greater water accessibility. At both reporter sites in MMs, these osmotic volume changes were nearly identical, but their spectral shapes and positions still differ. Hence, the two cysteine residues still sample their differently hydrated local water pockets but may nevertheless participate allosterically in the same osmotically induced overall volume change since the slope of the regression lines obtained for both labels is almost identical.

In DDM micelles, by contrast, the osmotic responses of the two reporters diverged markedly. BADAN at C382 reported approximately twice the exchangeable hydration volume detected at C384. Thus, the collective hydration response observed in MMs appears to become partially uncoupled in DDM, with the two residues reporting more independent hydration domains around the CBS.

NDs showed lower basal hydration and smaller osmolyte-induced changes. This indicates that reconstitution into NDs already reduces internal hydration of LpCopA, leaving less osmotically exchangeable water within a more compact transmembrane domain. Consistent with this interpretation, the BADAN emission spectra in NDs were more blue-shifted than in detergent-based systems even in the absence of osmolyte. This behavior agrees with our earlier observations for LpCopA reconstituted in asolectin-containing bilayers [18].

PEG-200 experiments were designed to examine whether the two reporter sites differ in their local response to a smaller osmolyte. Low-molecular-mass osmolytes can access protein cavities or channels that remain inaccessible to larger polymers such as PEG-1500 [19]. Whereas BADAN at C382 was largely insensitive to PEG-200 in NDs, the red shift observed at C384 suggests penetration of the smaller osmolyte into the BADAN binding site or an osmolyte-induced rearrangement of local hydration water without net dehydration. Similar effects of PEG-200 have been observed for rhodopsin [28].

Despite this ambiguity, the result evidences that the presence of a planar bilayer restructures the coupling of the CBS polarity to the size of water-filled crevices that communicate with the aqueous phase.

DIBMALPs exhibited the smallest osmotic response and the lowest basal hydration of all systems tested, consistent with the most compact transmembrane packing. Notably, this behavior differed markedly from that in NDs, although both systems provide discoidal lipid bilayers. The properties of the surrounding scaffold therefore appear to influence the packing and hydration of LpCopA beyond the presence of a nominally planar lipid bilayer alone. NDs and DIBMALPs were of very similar size and lipid to protein ratio. This renders a direct interaction of DIBMA with LpCopA very unlikely. Instead, we attribute the “stiffness” of the LpCopA structure in DIBMALPs to an increased lateral pressure exerted on the lipid bilayer as compared to that in NDs.

The lipid composition of the membrane mimetics must also be considered when interpreting these differences. Both MMs and NDs were prepared using the same *E. coli* polar lipid extract, which contains phosphatidylethanolamine (PE), phosphatidylglycerol (PG) and cardiolipin (CL) as major phospholipid classes. In contrast, DIBMA extracted LpCopA together with lipids directly from the *E. coli* inner membrane. The *E. coli* membrane is likewise dominated by PE, PG and CL [29]. The major fatty acyl constituents of *E. coli* phospholipids include C14:0, C16:1, C16:0, cyC17:0 and C18:1 [30]. Thus, while the precise local lipid environment surrounding LpCopA was not determined in any of the membrane mimetics, their common basis in *E. coli*-derived phospholipids suggests that the pronounced differences in BADAN response are unlikely to be explained by lipid composition alone, instead pointing to an important contribution of the physical organization of the surrounding lipid phase which modulates the lateral bilayer compressibility.

Although the necessarily simplified analysis of static BADAN emission does not capture the full distribution of fluorescent microstates or the time-dependent decrease in photon energy during dipolar relaxation, the overall trend is clear. Relative to detergent-based systems, bilayer-based reconstitution in NDs and especially in DIBMALPs reduces internal hydration of the transmembrane domain. Together, these observations support the conclusion that the membrane environment modulates both local hydration near the CBS and the larger osmotically exchangeable volume of the entire LpCopA structure.

### 3.2. Internal Hydration Is Associated with Catalytic Activity

Across the lipid-containing reconstitution systems, ATPase activity followed the amount of osmotically exchangeable water: activity and hydration were highest in MMs, intermediate in NDs, and strongly reduced in DIBMALPs. The specific ATPase activity of 53.5 ± 4.0 nmol P_i_ mg^−1^ min^−1^ measured in MMs was in the same range as the value of 65.0 ± 5.0 nmol P_i_ mg^−1^ min^−1^ previously reported for LpCopA in *E. coli* lipid-containing micelles [15], supporting the use of the MM condition as a functionally competent reference state. This association suggests that a certain degree of basal internal hydration and conformational flexibility facilitates catalytic turnover.

A strong dependence of P-type ATPase function on the physical properties of the surrounding lipid phase has previously been demonstrated for SERCA. Reconstitution into short-chain C14:1 phosphatidylcholine reduced Ca^2+^ binding from approximately two to one ion per ATPase molecule, whereas two ions per ATPase binding stoichiometry was retained in C18:1 phosphatidylcholine [31]. In the same short-chain C14:1 lipid environment, ATPase activity and phosphoenzyme formation were strongly reduced. Decreased activity as well as altered Ca^2+^ binding could partly be reversed by addition of the hydrophobic membrane additive androstenol [32]. Here, we have shown that an ensemble property of the lipidic phase, i.e., lateral pressure, is a crucial factor that needs to be taken into account in functional studies using membrane mimetics.

Pure DDM was an important exception to the observed internal hydration–activity correlation, because it supported substantial osmotic responsiveness but little catalytic activity. Functional impairment of membrane proteins in pure detergent has been described extensively before [15,33,34,35] and is commonly attributed to the loss of essential protein–lipid interactions. Studies of sarcoplasmic reticulum Ca^2+^-ATPase indicate that detergent-induced loss of activity can additionally arise from direct perturbation of specific steps within the catalytic cycle. Importantly, these effects were already observed at non-solubilizing DDM concentrations: DDM reduced the affinity of the second high-affinity Ca^2+^-binding site and abolished cooperative Ca^2+^ binding and, more prominently, the subsequent E1P-to-E2P isomerization [36,37]. Related work further showed that phospholipids stabilize detergent-solubilized Ca^2+^-ATPase even when the protein remains monomeric, arguing that lipid–protein and detergent–protein interactions, rather than loss of protein–protein contacts alone, are major determinants of stability and turnover [38]. Thus, the pronounced hydration response observed for LpCopA in DDM does not necessarily imply preservation of a catalytically competent conformational cycle. The data therefore point to a combined requirement for specific lipid interactions and a membrane environment that permits hydration-linked structural rearrangements.

In contrast, the low ATPase activity in DIBMALPs, together with the strongly reduced osmotic response, is consistent with an elevated level of transmembrane compaction that restricts the hydration-mediated conformational flexibility required for catalytic turnover. Such compaction does not require direct interaction between the DIBMA polymer and LpCopA. Rather, the polymer appears to exert an unusually large lateral pressure on the lipid bilayer which is transmitted to the ATPase. In comparison to membrane scaffold protein NDs, distinct lipid dynamics in DIBMALPs have also been reported [39], as well as altered lipid ordering and water accessibility in contrast to liposomes [40].

A conceptually similar effect has been proposed for amphipols, which reversibly inhibit the ATPase activity of SERCA1a. This behavior has been attributed to a “Gulliver effect”, in which multipoint interactions between the amphipathic polymer and the hydrophobic transmembrane surface dampen collective movements of the helix bundle and thereby restrict the “breathing” motions required during the catalytic cycle [41]. Furthermore, close adaptation of amphipathic polymers to membrane-protein surfaces has previously been observed in a cryo-EM structure of a supercomplex comprising alternative complex III and cytochrome oxidase solubilized with styrene malic acid copolymer [42]. Restrictions in membrane proteins reconstituted in amphipol-based mimetics are proposed to arise from direct polymer–protein contact, whereas in DIBMALPs the present data are consistent with an indirect effect mediated through bilayer lateral pressure.

### 3.3. Mechanistic Aspects of Hydration-Dependent Catalytic Activity of LpCopA

The dependence of ATPase activity on the reconstitution system has direct mechanistic implications, particularly when comparing MMs and NDs. The rate of ATP hydrolysis was about 43% higher in MMs than in NDs, suggesting that a planar bilayer imposes higher energy barriers along the trajectory of functionally required conformational changes of LpCopA than the much less constrained and more flexible MMs. We hypothesize that membrane-dependent differences in conformational flexibility and hydration-linked transmembrane rearrangements contribute to the energetic barriers of the catalytic cycle. This is in line with the reduction of internal hydration of LpCopA by either osmotic stress on the MM or by reconstitution into NDs, which themselves exert a dehydrating effect upon compaction of the transmembrane helical bundle.

In order to estimate the apparent free energy difference Δ*G*_cmp_ for compacting the LpCopA structure from the more hydrated MM state to the less hydrated ND state, we compared the internal hydration equilibria in the absence of osmolytes. The Gaussian band intensity ratios of 0.36 in MMs and 0.84 in NDs for BADAN@C382 (Figure 6) correspond to an apparent free energy difference of approximately 2 kJ mol^−1^ between these states. Independently, the osmolyte experiments indicated that the MM and ND states differ by an osmotically exchangeable water volume of approximately 500 Å^3^. Assuming a bilayer thickness of 5 nm and approximating the transmembrane helical bundle as a cylinder, this volume difference corresponds to an effective cross-sectional area change Δ*A*_cs_ of the TM domain of approximately 10 Å^2^ against lateral pressure. This simplified model cannot account for the full lateral pressure profile [16] but provides a plausible approximation of the mechanical energy of compaction Δ*G*_cmp_ in the form of

(2) $$∆G_{cmp}=∆A_{cs}\cdot ∆p_{lat},$$

where Δ*p*_lat_ denotes the difference in bilayer-imposed lateral pressure between MMs and NDs. Attributing the entire apparent free-energy difference of approximately 2 kJ mol^−1^ to this contribution yields a lateral-pressure equivalent of 33 mN m^−1^ upon transfer of LpCopA from MMs into NDs. However, the transition from MMs to NDs also changes membrane curvature, lipid-chain ordering and bilayer thickness. Consequently, Δ*G*_cmp_ may include contributions from curvature-dependent deformation, hydrophobic mismatching, and other membrane–protein interactions that are not explicitly represented by Equation (2). Nevertheless, its magnitude is comparable to the equivalence surface pressure between lipid monolayers and bilayers of 35 mN m^−1^ [43]. Conversely, there is virtually no lateral pressure in MMs as expected in the absence of a planar bilayer and the lack of a polymeric envelope in MMs.

In an analogous way, the difference in catalytic rates between the two reconstitutions can be expressed as an apparent activation free energy difference of ΔΔ*G^#^* ≈ 0.9 kJ mol^−1^. If this kinetic difference is interpreted within the same simplified mechanical framework introduced above, and using the effective stress scale of Δ*p*_lat_ ≈ 33 mN m^−1^, it corresponds to a cross-sectional area change Δ*A*_cs_ of approximately 4.5 Å^2^.

Thus, both analyses show that relatively small hydration-linked changes in transmembrane packing may accompany different functional and catalytic transition states supported by the two membrane mimetics. Assuming a cross-sectional area of ~1000 Å^2^ for the octahelical TM bundle of LpCopA, the determined cross-sectional area changes correspond to only about 0.1 and 0.05 Å change in diameter for LpCopA in MMs versus NDs and the LpCopA ground state versus the LpCopA transition state in NDs, respectively. Figure 6 summarizes the derived energetic and geometrical quantities.

Taken together, the osmolyte experiments revealed the presence of exchangeable intramembrane water in LpCopA. Hydration was not uniform around the CBS, as C382 and C384 differed in their basal environments and, depending on the membrane mimetic, also in their coupling to osmotic stress. The amount of exchangeable water varied markedly among the reconstitution systems and was highest in MMs, reduced in NDs, and nearly absent in DIBMALPs. These differences paralleled the catalytic behavior of the unlabeled protein, with MMs supporting the highest ATPase activity, NDs intermediate activity, and DIBMALPs markedly reduced activity. This basal hydration appears to be required for function. Exchange of a fraction of the hydration water with the aqueous phase during catalytic turnover would entail changes in the TM diameter of only a few percent of an Å against lateral pressure. Therefore, even such a small expansion may contribute to the transition state energy of LpCopA. Differences in bilayer lateral pressure can thus become a crucial factor in the functional studies of membrane proteins in planar membrane mimetics.

## 4. Materials and Methods

### 4.1. Overproduction and Purification of LpCopA

Wild-type LpCopA as well as LpCopA mutants (single-cysteine mutants BADAN@C382 and BADAN@C384, and the cysteine-free mutant, cf) were produced and purified as previously described [18]. Additional details are provided in the Supporting Information.

### 4.2. Site-Directed Labeling of LpCopA

To obtain site-specific information on the polarity of the active-site microenvironment, the fluorescence probe 6-bromoacetyl-2-dimethylaminonaphthalene (BADAN) was covalently attached to either cysteine residue of the CPC motif on helix M4. Nonspecific labeling was minimized by employing an on-column labeling strategy. Protein expression, cell lysis, solubilization, and centrifugation were performed as described in the Section 4.1. section titled *Overproduction and purification of LpCopA*.

The sample was adjusted to 50 mM imidazole and loaded onto a 1 mL Ni-NTA HisTrap column equilibrated with KT buffer (50 mM Tris, pH 7.4, 200 mM KCl, 1 mM MgCl_2_, and 10% glycerol) supplemented with 0.25% (*w*/*v*) DDM and 50 mM imidazole. Bound protein was labeled by washing with 15 column volumes (CVs) of BADAN wash buffer (50 mM imidazole, 0.25% (*w*/*v*) DDM, 15 µM BADAN dissolved in KT buffer) at a flow rate of 0.3 mL min^−1^. Labeling was followed by extensive washing with 70 CVs of equilibration buffer and 20 CVs of KT buffer containing 0.15% (*w*/*v*) DDM and 50 mM imidazole. The protein was eluted using a linear imidazole gradient from 50 to 500 mM. PD-10 desalting columns (Cytiva, Fairfield, CT, USA) were used to exchange the labeled protein into buffers suitable for downstream applications.

### 4.3. Overproduction and Purification of Membrane Scaffold Protein

The membrane scaffold protein variant MSP1E3D1 was used for nanodisc preparation. The plasmid pMSP1E3D1 was obtained from Addgene (plasmid #20066) (Watertown, MA, USA). Details of MSP1E3D1 overproduction and purification are provided in the Supporting Information.

### 4.4. Preparation of DIBMA

DIBMA polymer powder (Acusol 460ND, kindly provided by the group of Prof. Sandro Keller) was dissolved in water to a final concentration of 200 mg mL^−1^. Dissolution was facilitated by alternating incubation in a 60 °C water bath and vortexing. Three milliliters of the DIBMA solution were dialyzed against 1 L of buffer A (50 mM Tris, pH 7.4, 200 mM NaCl) using a 3.5 kDa MWCO dialysis membrane (Spectrum Laboratories, Inc., Rancho Dominguez, CA, USA) at room temperature for a total of 16 h, with buffer replacement after 8 h. After dialysis, the solution was filtered through a 0.22 µm filter. To enable accurate determination of membrane-to-polymer mass ratios, the dialyzed DIBMA solution was lyophilized using a SpeedVac (Thermo Scientific GmbH, Waltham, MA, USA). The resulting DIBMA powder was stored at 4 °C.

### 4.5. Assembly and Purification of LpCopA in MSP1E3D1 Nanodiscs

LpCopA or its mutants were exchanged into HNMS buffer (50 mM HEPES, pH 7.4, 200 mM Na_2_SO_4_, 5 mM MgSO_4_) containing 0.1% (*w*/*v*) DDM and 3 mM BME using PD-10 columns and subsequently concentrated using Macrosep Advance centrifugal devices (30 kDa MWCO) (Pall Corporation, New York, NY, USA). *E. coli* polar lipid extract (Avanti Polar Lipids, AL, USA) was dissolved to a concentration of 4 mM in water containing 12 mM DDM. The lipid solution was vortexed and sonicated until homogeneous.

LpCopA, MSP1E3D1, and lipids were mixed at a molar ratio of 1:6:200 (3–7 µM CopA) and incubated on ice for 1 h. Detergent removal was performed using detergent removal spin columns (Thermo Scientific GmbH, Waltham, MA, USA). The flow-through was adjusted to 20 mM imidazole and applied to a HisTrap spin column (Cytiva, Fairfield, CT, USA) to separate empty lipid nanodiscs from protein-containing nanodiscs. Elution fractions were pooled, centrifuged at 20,000× *g* for 10 min at 4 °C, and further purified by size-exclusion chromatography using a Superdex 200 Increase 10/300 GL column (Cytiva, Fairfield, CT, USA) to obtain monodisperse nanodiscs (Figure S1).

### 4.6. Formation of LpCopA-Containing DIBMA Lipid Particles

DIBMA enables direct solubilization of native membranes together with their intrinsic membrane proteins [44]. Accordingly, DIBMA was added to membranes isolated from CopA-overproducing *E. coli* strains. Cell culture, lysis, and membrane extraction were performed as described above, except that the lysis buffer contained 50 mM Tris and 200 mM NaCl supplemented with protease inhibitors, DNase I, and lysozyme. The membrane pellet was weighed, disrupted using a Dounce homogenizer, and adjusted to a concentration of 60 mg mL^−1^ with lysis buffer.

DIBMA was added at a mass ratio of 0.83 (*m*_DIBMA_/*m*_wet membrane_), and the mixture was incubated for 2 h at room temperature with agitation. Insoluble material was removed by ultracentrifugation at 100,000× *g* for 1 h. In parallel, 2 mL of PureCube Ni-NTA agarose (Cube Biotech, Monheim, Germany) were equilibrated in a spin column with buffer containing 50 mM Tris, 200 mM NaCl, 10 mM imidazole, and 3 mM BME. The solubilized sample was incubated with the resin overnight at room temperature on an end-over-end shaker. After batch binding, the flow-through was collected, followed by four wash steps (50 mM Tris, 200 mM NaCl, 20 mM imidazole, 3 mM BME). His-tagged LpCopA DIBMALPs were eluted stepwise using increasing imidazole concentrations (250, 300, 450, and 500 mM; 2 mL each). PD-10 columns were used for imidazole removal. To eliminate aggregates, the eluate was centrifuged at 100,000× *g* for 1 h at 4 °C (Figures S2 and S3).

### 4.7. BADAN Labeling of LpCopA in E. coli Membranes Prior to DIBMA Solubilization

Because direct formation of DIBMALPs precludes on-column labeling of detergent-solubilized LpCopA, BADAN was added directly to *E. coli* membranes containing overexpressed LpCopA. The membrane harvesting step was performed as described above. This was followed by extensive washing and dilution with additional *E. coli* lipids prior to DIBMA-mediated solubilization. Cell culture, lysis, and membrane extraction were carried out as described above. Membranes were resuspended using a Dounce homogenizer, and BADAN dissolved in DMF was added to a final concentration of 30 µM. The membrane-BADAN mixture was incubated for 30 min at 4 °C.

Membranes were pelleted by ultracentrifugation at 100,000× *g* for 1 h at 4 °C, resuspended in buffer by Dounce homogenization, and centrifuged again. This wash cycle was repeated four times to promote partitioning of unbound BADAN into the aqueous phase. The wet membrane mass was determined, and an equal mass of *E. coli* membranes lacking CopA was added. The combined membranes were solubilized with DIBMA at a mass ratio of 0.83 (*m*_DIBMA_/*m*_wet membrane_). Further processing of BADAN-labeled DIBMALPs was performed as described in the Section 4.6.

### 4.8. Preparation of LpCopA in C_12_E_8_-Based Mixed Micelles

LpCopA used for mixed micelle experiments was overproduced and purified following the same general procedure described for the DDM-solubilized protein (Section 4.1 and Supporting Information), except that C_12_E_8_ was used as detergent during membrane protein solubilization and purification. Following cell disruption and removal of cell debris, membrane proteins were solubilized with 0.8% (*w*/*v*) C_12_E_8_ for 40 min at 4 °C. After removal of unsolubilized material, LpCopA was purified by Ni-NTA affinity chromatography using buffers containing 0.15% (*w*/*v*) C_12_E_8_ during column loading, washing, elution, and desalting steps.

For site-specific BADAN labeling of the single-cysteine variants used in mixed micelles, the on-column labeling procedure described in Section 4.2 was followed, with 0.15% (*w*/*v*) C_12_E_8_ replacing DDM in the corresponding detergent-containing buffers.

For preparation of the final mixed-micelle samples, *E. coli* polar lipids were subsequently resuspended in 10 mM C_12_E_8_ to a concentration of 2 mg mL^−1^ and mixed until a clear solution was obtained. Purified LpCopA was thawed and buffer-exchanged using PD-10 desalting columns into ATPase assay buffer (50 mM HEPES, pH 7.0, 75 mM Na_2_SO_4_, 2 mM TCEP, and 5 mM MgSO_4_) containing 0.28% (*w*/*v*) C_12_E_8_. The solubilized lipid preparation was then added to obtain a final lipid concentration of 0.14 mg mL^−1^ at an LpCopA concentration of 0.20 mg mL^−1^. Samples were mixed for 15 min at room temperature and for 15 min at 4 °C and subsequently centrifuged at 20,000× *g* for 10 min at 4 °C to remove aggregates. The protein concentration in the clarified supernatant was determined spectrophotometrically before further use.

### 4.9. ATPase and Malachite Green Assay

During copper transport, ATP is hydrolyzed to ADP and inorganic phosphate. The amount of released phosphate was quantified using a colorimetric malachite green assay to assess ATPase activity [44,45,46]. Assay conditions were largely adapted from previous work [15]. CopA samples in different membrane-mimetic systems were exchanged into ATPase assay buffer (50 mM HEPES, pH 7.0, 75 mM Na_2_SO_4_, 2 mM TCEP, and 5 mM MgSO_4_) using PD-10 columns. LpCopA in mixed micelles was prepared as described in Section 4.8.

AgNO_3_ was added to a final concentration of 500 µM, and samples were incubated at 37 °C. Reactions were initiated by addition of 5 mM ATP disodium salt hydrate (pH 7.0). At defined time points (0, 5, 10, 20, and 30 min), aliquots (160 µL) were quenched by rapid freezing in liquid nitrogen. Phosphate quantification was based on formation of a phosphomolybdate complex, followed by malachite green intercalation. Strict timing of pipetting steps was essential: at *t* = 0 min, 50 µL of 20% trichloroacetic acid were added, samples were thawed at 70 °C for 15 s, and vortexed. At *t* = 2 min, samples were centrifuged for 1 min at 13,200 rpm. At *t* = 3.5 min, 60 µL of supernatant were transferred into tubes containing 100 µL acidic molybdate solution. At *t* = 3.75 min, 30 µL malachite green solution were added. At *t* = 5.75 min, free malachite green was degraded by addition of 200 µL 7.8% H_2_SO_4_. After 70–100 min of color development, 200 µL were transferred to a 96-well plate and absorbance was measured at 625 nm.

### 4.10. Sample Preparation for Osmolyte Experiments

Stock solutions of 50% (*w*/*v*) PEG were prepared in 50 mM HEPES, 75 mM Na_2_SO_4_, 5 mM KNO_3_, 5 mM MgSO_4_, and 2 mM TCEP at pH 7.0 and filtered through 0.22 µm sterile filters. Stock solutions were mixed with protein samples to yield final osmolyte concentrations between 2.5% and 32.5%. Samples were equilibrated for 15 min at 20 °C under constant agitation prior to fluorescence measurements.

Osmotic pressure was calculated for the corresponding PEG concentrations using the following polynomial expression [22]:

(3) $$\pi \;=\;RT\left(\frac{10}{M}c\;+\;Ac^{2}\;+\;Bc^{3}\right),$$

where *M* is the molecular mass of the osmolyte and *c* (g dL^−1^) is the osmolyte concentration. The second and third virial coefficients for PEG-1500 were taken as *A* = 4.7 × 10^−4^ and *B* = 0.9 × 10^−5^, with units defined as *A* = *a*(10/*M*)^2^ and *B* = *b*(10/*M*)^3^ [22]. PEG concentrations were converted from g per 100 g to g dL^−1^ using the density δ of PEG solutions at 298 K, which depends on the mass fraction *w* (g g^−1^) but not on PEG molecular mass [47]:

δ = 997.07 + 114.41*w* (4)

### 4.11. Fluorescence Measurements

Steady-state fluorescence spectra of LpCopA in different osmolyte solutions were recorded using an LS 55 luminescence spectrometer (PerkinElmer, Shelton, CT, USA). Excitation was set to 385 nm with a bandwidth of 5 nm for both excitation and emission. Spectra were collected at a scan speed of 100 nm min^−1^.

### 4.12. Gaussian Band Decomposition of Emission Spectra

BADAN emission spectra were analyzed by Gaussian band decomposition over the wavelength range from 420 to 515 nm, which corresponds to the symmetrically extended range around the peak position. The lower wavelength limit was chosen to avoid interference with the excitation band at 385 nm. Each spectrum was reconstructed as the superposition of two Gaussian components. The amplitude, peak position, and full width at half maximum (FWHM) of both components were allowed to vary independently for each spectrum. No fixed peak positions or widths were imposed across osmolyte concentrations or membrane-mimetic systems, thereby allowing the fitting procedure to account for shifts of the overall emission contour upon osmolyte addition.

The two Gaussian components were treated as empirical representations of weakly and strongly hydrated emitting ensembles. Their integrated areas, a1 and a2, were used as proxies for their relative spectral contributions, and an equilibrium parameter was defined as

(5) $$K=\frac{a1}{a2}.$$

The osmolyte shifts the molar free energy Δ*G* of the equilibrium between the weakly and strongly hydrated ensembles by the amount Δ*V*_m_ which is obtained from

(6) $$\mathrm{RT}\;\frac{\partial \;ln\;K}{\partial \pi }$$

where *R* is the universal gas constant, *T* the absolute temperature, and π the osmotic pressure. The absolute value of *K* is not required but is estimated to be *K_w_ =* 1 for the conformational transition in the absence of osmolytes (further description is found in the Supporting Information).

To examine whether the spectral representation affected the Gaussian decomposition, selected BADAN emission spectra were additionally transformed to wavenumber space and analyzed analogously. The resulting Gaussian decomposition yielded comparable osmolyte-dependent behavior to the wavelength-based analysis (Figure S8).

## 5. Conclusions

Different membrane mimetics containing *E. coli*-derived lipids strongly modulate both the hydration state of the transmembrane domain of LpCopA and its catalytic activity. Mixed micelles supported the largest amount of osmotically exchangeable internal water and the highest ATPase activity; membrane scaffold protein nanodiscs exhibited intermediate levels of both, whereas DIBMALPs were consistent with the most compact and least osmotically responsive state and lowest catalytic turnover.

The results support an association between internal hydration, transmembrane packing, and catalytic activity. The energetic estimates further suggest that relatively small hydration-linked structural changes may contribute to the free energy associated with conformational transitions of LpCopA. In this view, internal hydration is not merely a passive structural feature but a functionally and kinetically important part of the energetic landscape of the transport cycle.

Future work will be required to resolve how these hydration changes map onto specific conformational states of the Post–Albers cycle and how they relate to ion binding, release, and transmembrane rearrangement.

## Figures and Tables

**Figure 1:**
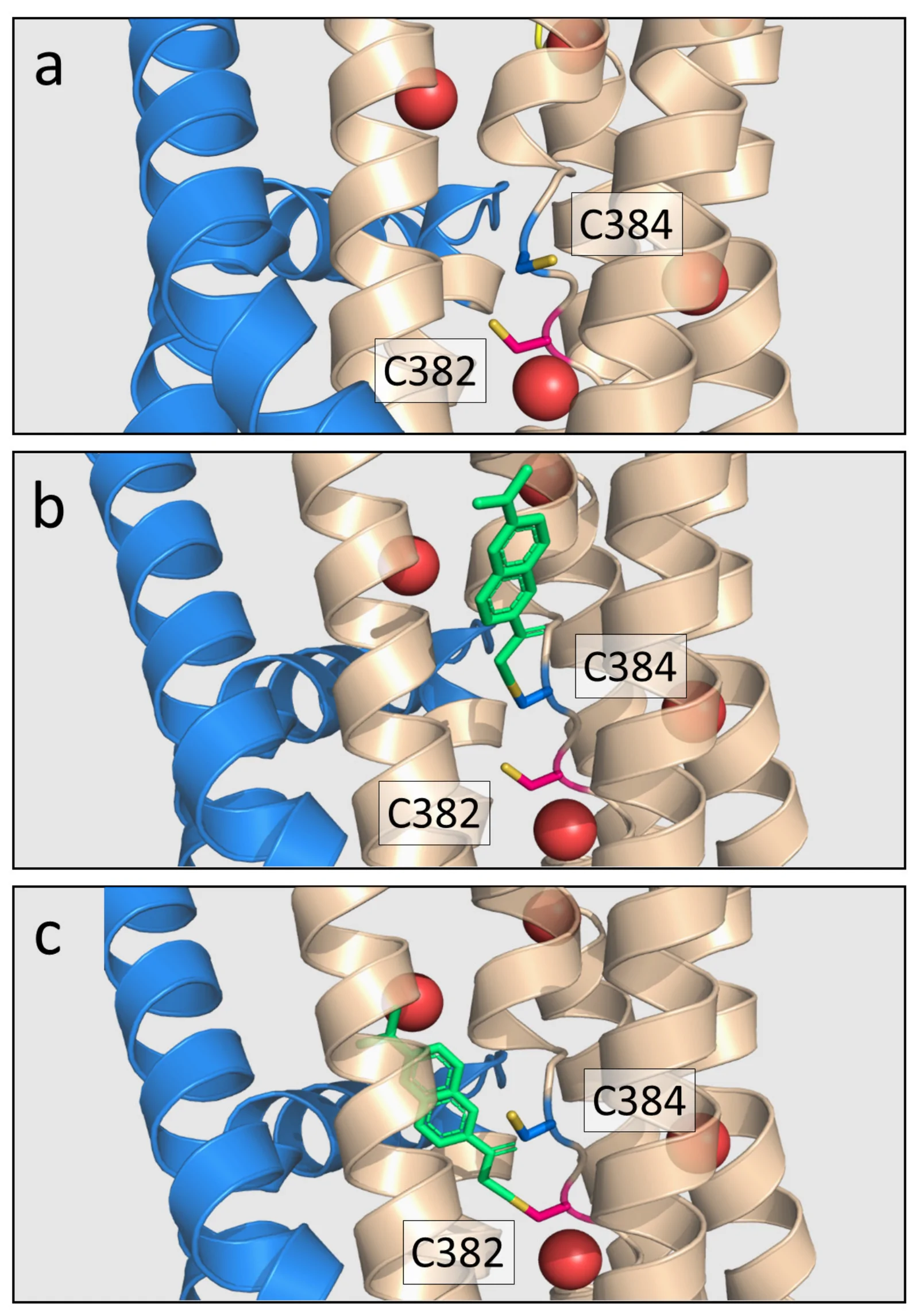
Schematic view of the canonical metal-binding site in the crystal structure of LpCopA (PDB 4BBJ) with highlighted cysteines (C382 and C384) and water molecules. (**a**) Wild-type LpCopA. (**b**) Hypothetical orientation of the fluorophore BADAN covalently linked to C384, designated BADAN@C384. (**c**) The equivalent model for BADAN@C382. The BADAN orientations are illustrative and are not intended to represent energy-minimized resolved conformations of the fluorophore. The representation shows the correct size of BADAN in the water-accessible binding pocket of LpCopA. Cysteines are in stick representation, whereas water molecules are displayed as red spheres.

**Figure 2:**
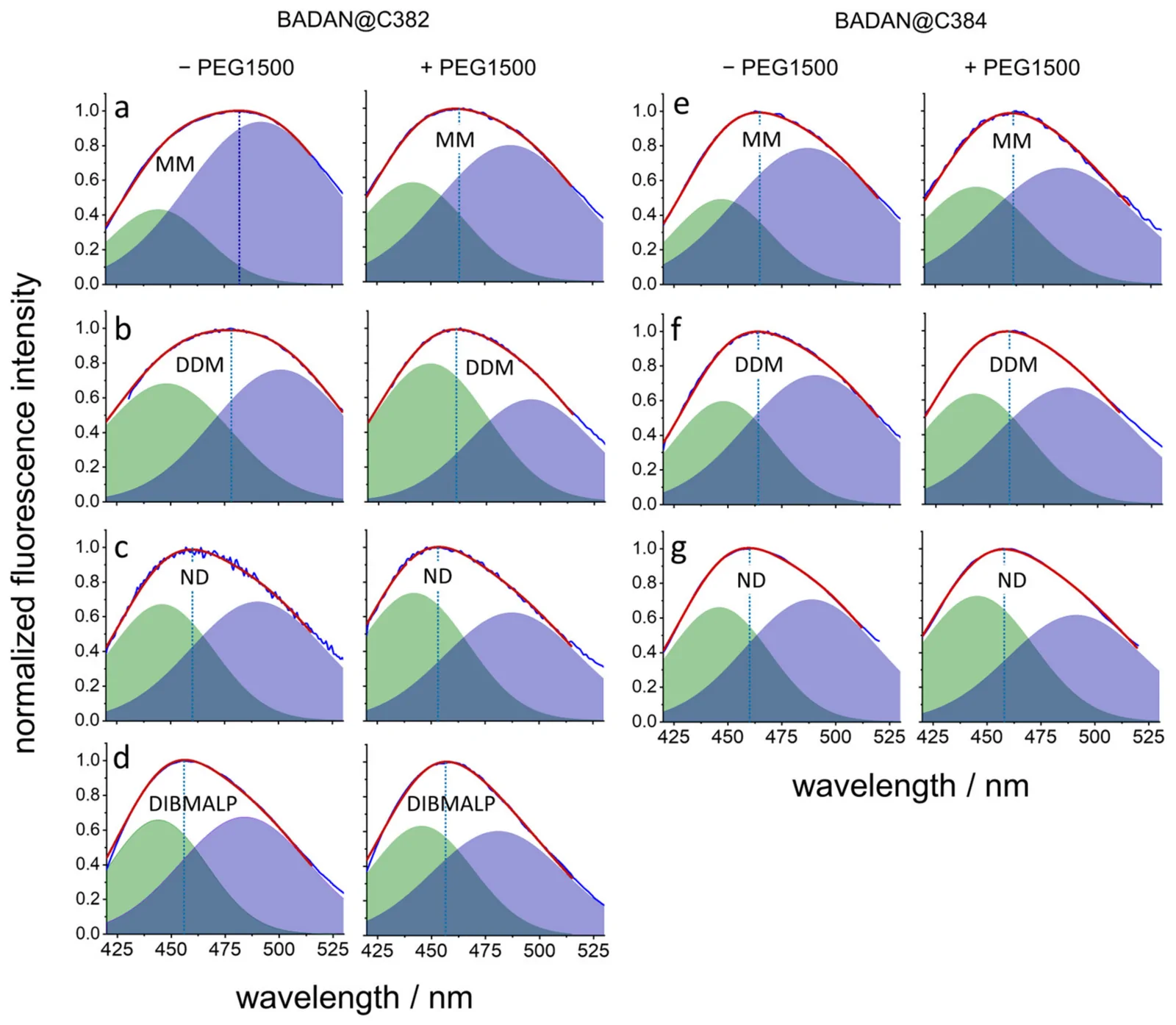
Gaussian decomposition of steady-state fluorescence spectra of BADAN-labeled single-cysteine LpCopA mutants in different membrane mimetic systems with and without PEG-1500. Left panel: (**a**–**d**) Fluorescence of BADAN bound to C382 in the indicated reconstitution systems, *n*-dodecyl-β-D-maltopyranoside (DDM) micelle, mixed micelle (MM), membrane scaffold protein nanodisc (ND), and diisobutylene-maleic acid lipid particle (DIBMALP). The spectra were recorded in aqueous buffer without or with 32.5% PEG-1500 as indicated at the top of the panel. Emission maxima are indicated by vertical, blue dotted lines. The recorded spectra (blue lines) were reproduced by the sum of two Gaussian bands (red lines) whose integrals were assigned to weakly hydrated (green areas) or strongly hydrated (blue areas) populations. Right panel: (**e**–**g**) The equivalent data obtained with BADAN bound to C384. In DIBMALPs, the osmotic-stress analysis was restricted to BADAN@C382.

**Figure 3:**
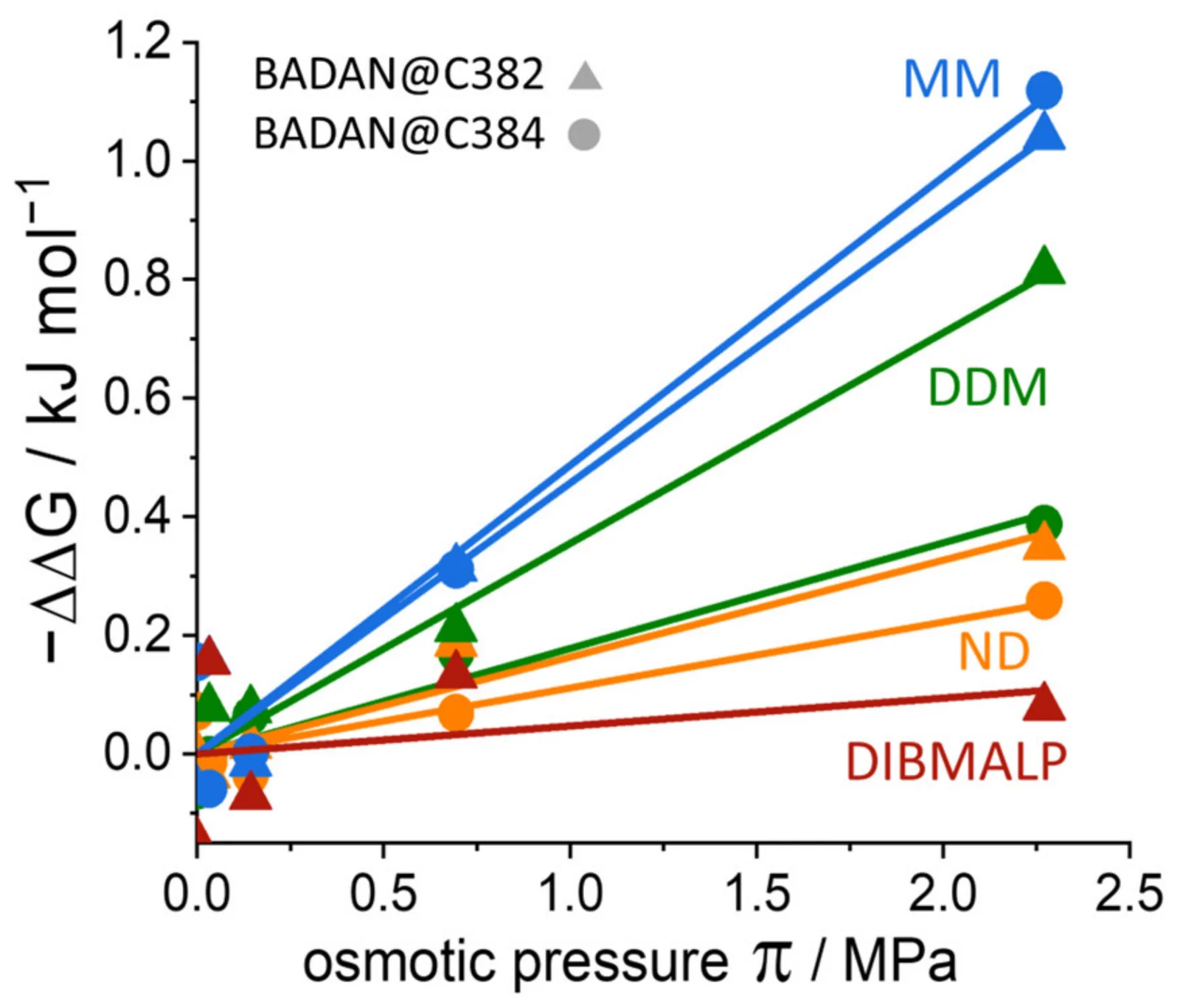
Free energy change ΔΔ*G* of the equilibrium between the strongly and weakly hydrated state as a function of osmotic pressure. Emission bands of BADAN-labeled CopA single cysteine mutants (BADAN@C382, triangles; BADAN@C384, filled circles) in MMs (blue), DDM micelles (green), NDs (orange) and DIBMALPs (red) were fitted by two Gaussian bands (Figure 2). The hydration equilibrium constant was defined as the ratio of the areas under the Gaussian bands for each osmotic pressure. The slopes of the linear fits provide the value of the molar volume change −Δ*V*_m_ upon osmotic dehydration. The derived amounts of exchanged water molecules are summarized in Table 1. In DIBMALPs, the osmotic-stress analysis was restricted to BADAN@C382. The molar free energy change between the strongly and weakly hydrated conformational states in the absence of osmolytes is estimated Δ*G* = 0 (further described in the Supporting Information).

**Figure 4:**
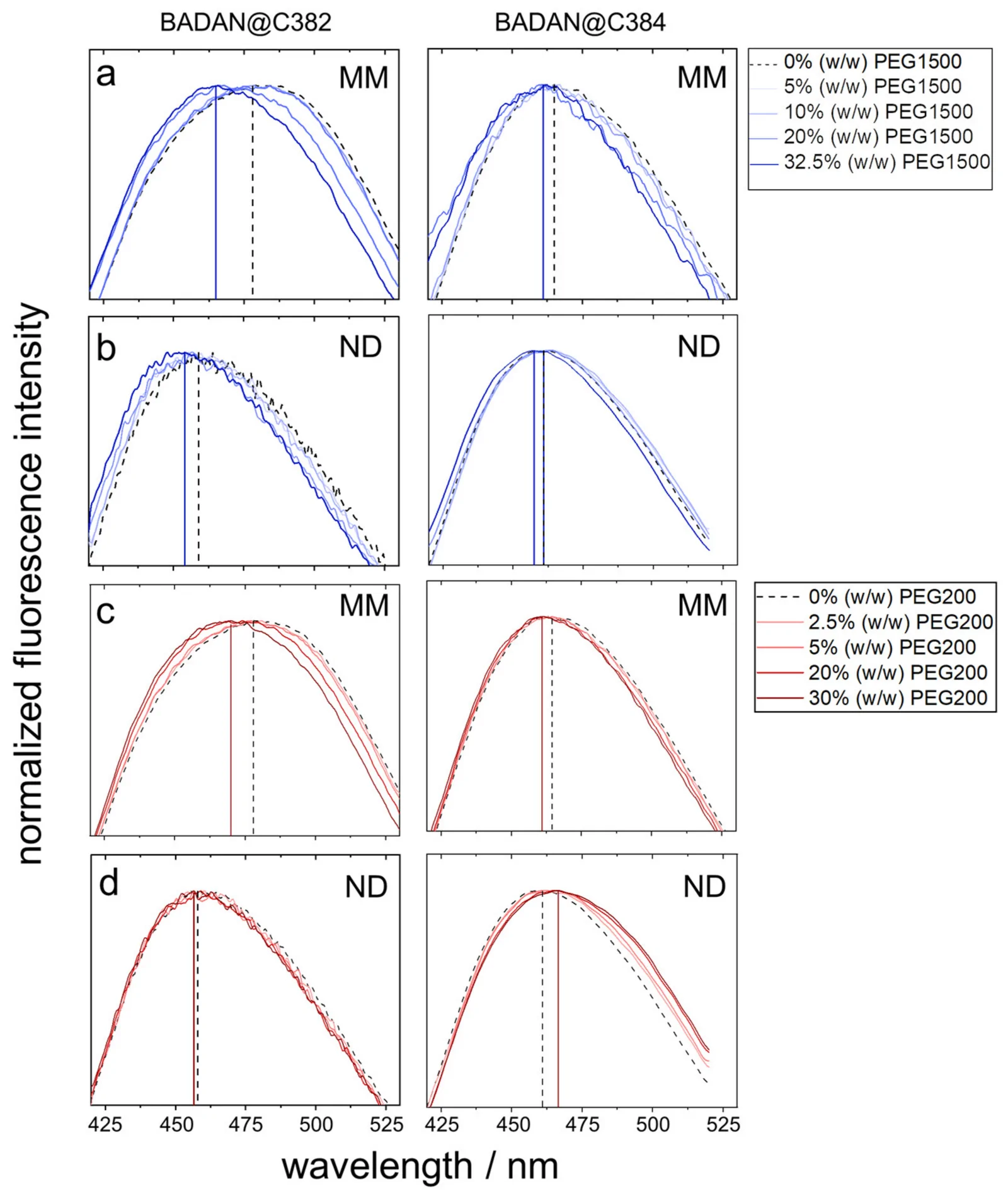
Osmolyte-dependent fluorescence response of BADAN-labeled LpCopA in mixed micelles (MM) and membrane scaffold protein nanodiscs (ND). Steady-state fluorescence spectra of BADAN@C382 and BADAN@C384 LpCopA recorded at increasing concentrations of PEG-1500 and PEG-200. (**a**) MMs probed with PEG-1500; (**b**) NDs probed with PEG-1500; (**c**) MMs probed with PEG-200; and (**d**) NDs probed with PEG-200. In each pair, the left panel shows BADAN@C382 and the right panel BADAN@C384. The complete spectral dataset for all membrane mimetics is provided in Figures S6 and S7.

**Figure 5:**
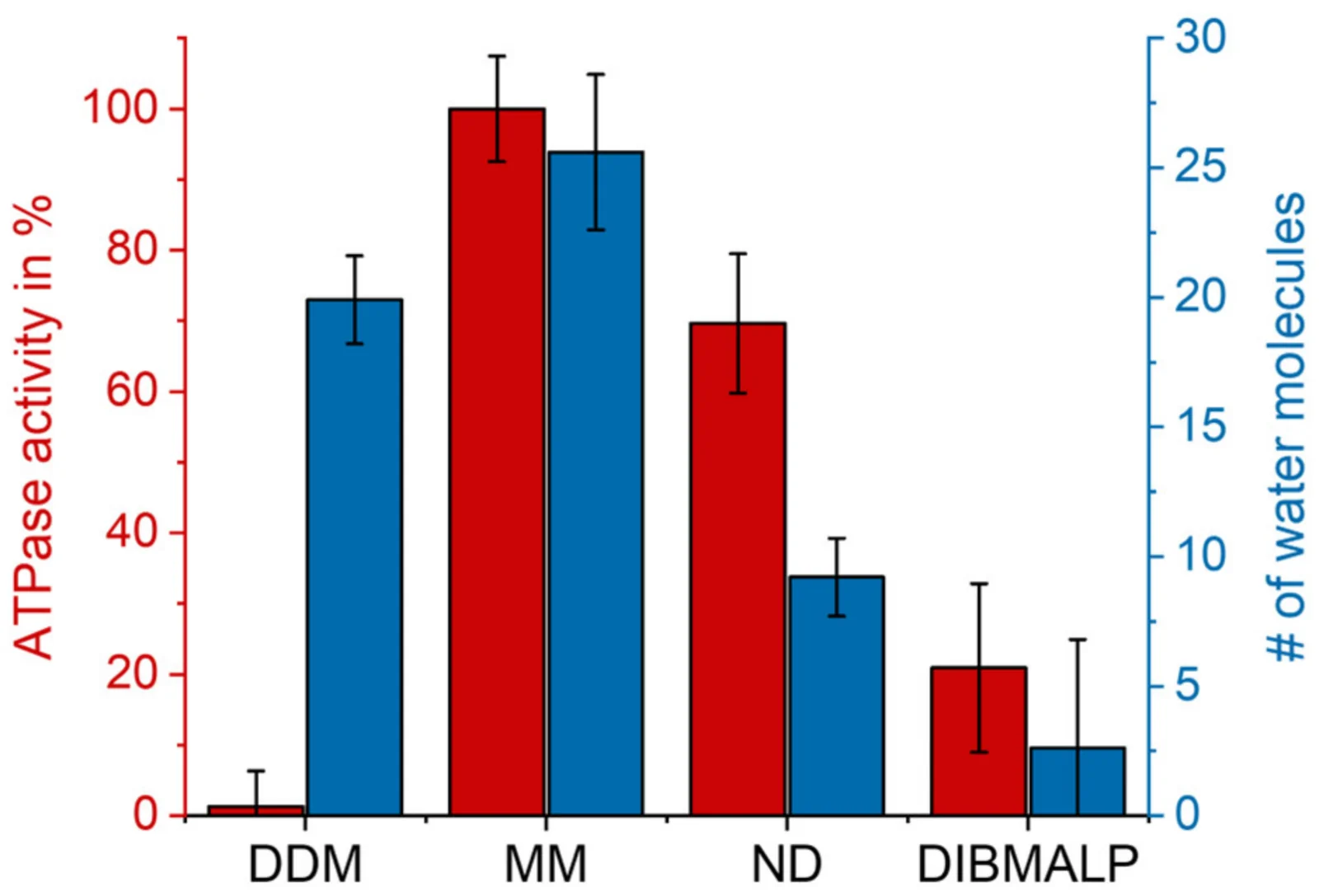
Comparison of the ATPase activity of wild-type LpCopA and osmotic hydration-linked volume change sensed through BADAN@C382 in different membrane mimetic systems. The ATPase activity of wild-type CopA as well as the site-specific hydration change of the BADAN@C382 mutant have been determined in the following membrane mimetic systems: DDM micelles, MMs, NDs and DIBMALPs; 100% activity corresponds to a value of 53.5 ± 4.0 nmol P_i_ mg^−1^ min^−1^. Further values for ATPase activity and number of water molecules can be found in Table 1.

**Figure 6:**
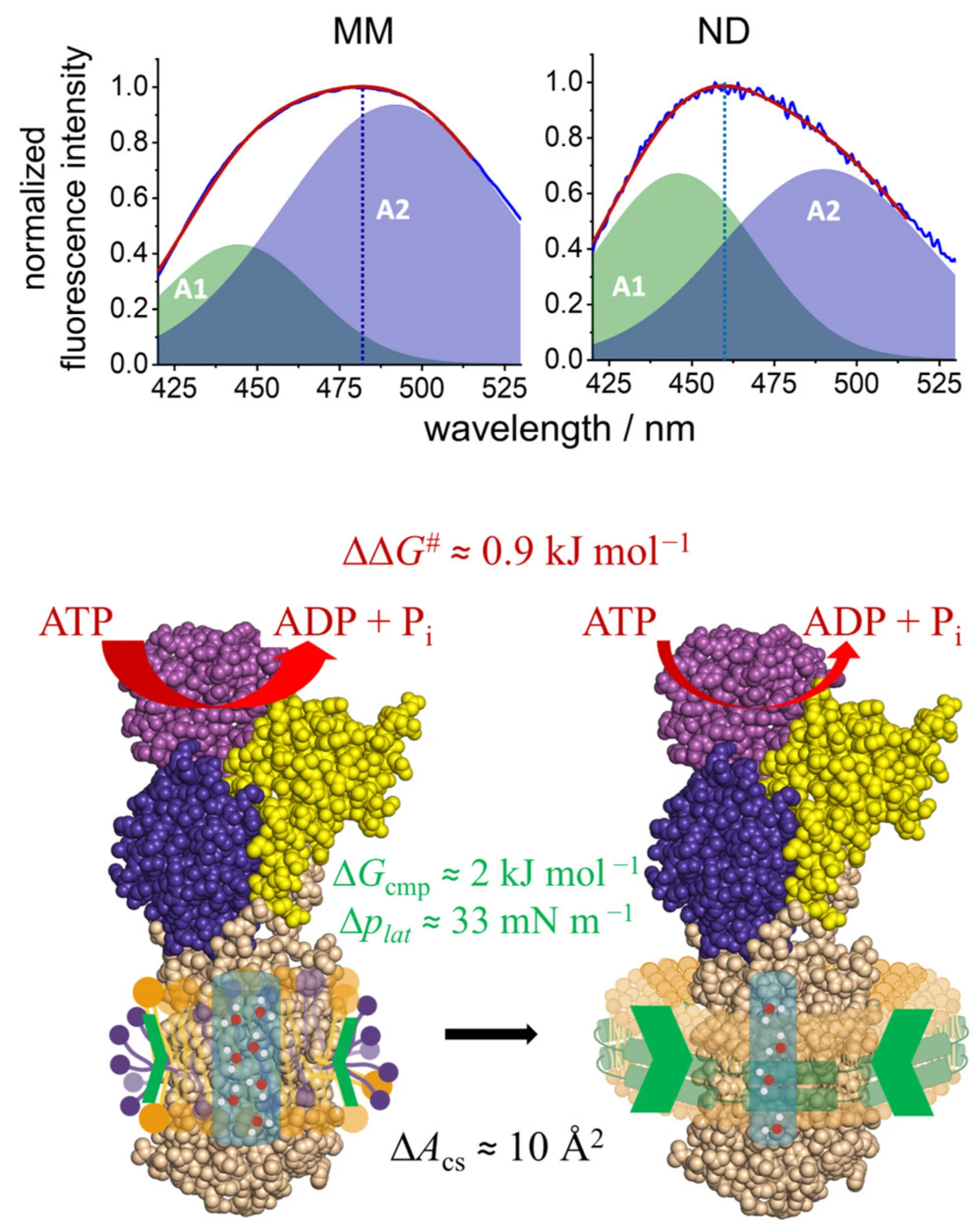
Estimated energetic and geometric differences between LpCopA reconstituted in mixed micelles (MM) and membrane scaffold protein nanodiscs (ND), which provide a bilayer environment. The ∆*G*_cmp_ between MMs and NDs has been derived from the change of the strongly hydrated (A2, blue area) to the weakly hydrated (A1, green area) population ratio in the emission spectra of BADAN linked to C382 (upper panel). The lateral pressure difference ∆*p*_lat_ between the membrane mimetics was calculated from ∆*G*_cmp_ for a 5 nm long cylindrical volume. ∆∆*G*^#^ was directly obtained from the ratio of catalytic rates in the two reconstitutions (Figure 5; Table 1).

## Data Availability

The raw data supporting the conclusions of this article will be made available by the authors on request.

## Supplementary Materials

The following supporting information can be downloaded at https://www.mdpi.com/article/10.3390/ijms27198870/s1.

## Abbreviations

The following abbreviations are used in this manuscript:

BADAN	6-bromoacetyl-2-dimethylaminonaphthalene
C_12_E_8_	octaethylene glycol monododecyl ether
CBS	canonical binding site
CL	cardiolipin
DDM	*n*-dodecyl-β-D-maltopyranoside
DIBMALP	diisobutylene-maleic acid lipid particle
LpCopA	*Legionella pneumophila* Cu^+^-ATPase
MM	mixed micelle
ND	membrane scaffold protein nanodisc
PE	phosphatidylethanolamine
PG	phosphatidylglycerol
PEG	polyethylene glycol
POPG	1-palmitoyl-2-oleoyl-*sn*-glycero-3-phosphoglycerol
SERCA	sarco(endo)plasmic reticulum Ca^2+^-ATPase

## References

[1] Pabst G., Keller S. (2024). Exploring membrane asymmetry and its effects on membrane proteins. Trends Biochem. Sci..

[2] Ji J., Lyman E. (2025). Lipid-GPCR interactions in an asymmetric plasma membrane model. Faraday Discuss..

[3] Doktorova M., Symons J.L., Zhang X., Wang H.-Y., Schlegel J., Lorent J.H., Heberle F.A., Sezgin E., Lyman E., Levental K.R. (2025). Cell membranes sustain phospholipid imbalance via cholesterol asymmetry. Cell.

[4] Cymer F., von Heijne G., White S.H. (2015). Mechanisms of integral membrane protein insertion and folding. J. Mol. Biol..

[5] Mulkidjanian A.Y., Galperin M.Y., Koonin E.V. (2009). Co-evolution of primordial membranes and membrane proteins. Trends Biochem. Sci..

[6] Møller J.V., Olesen C., Winther A.-M.L., Nissen P. (2010). The sarcoplasmic Ca^2+^-ATPase: Design of a perfect chemi-osmotic pump. Q. Rev. Biophys..

[7] Helmann J.D. (2025). Microbial metal physiology: Ions to ecosystems. Nat. Rev. Microbiol..

[8] Kim E.-H., Charpentier X., Torres-Urquidy O., McEvoy M.M., Rensing C. (2009). The metal efflux island of *Legionella pneumophila* is not required for survival in macrophages and amoebas. FEMS Microbiol. Lett..

[9] Rankin S., Li Z., Isberg R.R. (2002). Macrophage-induced genes of *Legionella pneumophila*: Protection from reactive intermediates and solute imbalance during intracellular growth. Infect. Immun..

[10] Andersson M., Mattle D., Sitsel O., Klymchuk T., Nielsen A.M., Møller L.B., White S.H., Nissen P., Gourdon P. (2014). Copper-transporting P-type ATPases use a unique ion-release pathway. Nat. Struct. Mol. Biol..

[11] Palmgren M. (2023). P-type ATPases: Many more enigmas left to solve. J. Biol. Chem..

[12] Dyla M., Kjærgaard M., Poulsen H., Nissen P. (2020). Structure and Mechanism of P-Type ATPase Ion Pumps. Annu. Rev. Biochem..

[13] Gourdon P., Liu X.Y., Skjorringe T., Morth J.P., Møller L.B., Pedersen B.P., Nissen P. (2011). Crystal structure of a copper-transporting PIB-type ATPase. Nature.

[14] Placenti M.A., Roman E.A., González Flecha F.L., González-Lebrero R.M. (2022). Functional characterization of *Legionella pneumophila* Cu^+^ transport ATPase. The activation by Cu^+^ and ATP. Biochim. Biophys. Acta Biomembr..

[15] Autzen H.E., Koldsø H., Stansfeld P.J., Gourdon P., Sansom M.S.P., Nissen P. (2018). Interactions of a Bacterial Cu(I)-ATPase with a Complex Lipid Environment. Biochemistry.

[16] Marsh D. (2007). Lateral pressure profile, spontaneous curvature frustration, and the incorporation and conformation of proteins in membranes. Biophys. J..

[17] Albers R.W. (1967). Biochemical aspects of active transport. Annu. Rev. Biochem..

[18] Fischermeier E., Pospíšil P., Sayed A., Hof M., Solioz M., Fahmy K. (2017). Dipolar Relaxation Dynamics at the Active Site of an ATPase Regulated by Membrane Lateral Pressure. Angew. Chem. Int. Ed..

[19] Parsegian V.A., Rand R.P., Rau D.C. (1995). Macromolecules and water: Probing with osmotic stress. Methods Enzymol..

[20] Menon C.S.K., Huber T., Thaller L.E., Struts A.V., Cheng E.W., Bachler Z.T., Perera S.M.D.C., Sakmar T.P., Brown M.F. (2026). Membrane protein hydration bridges polymer physics and biology. Biophys. J..

[21] Koehorst R.B., Spruijt R.B., Hemminga M.A. (2008). Site-directed fluorescence labeling of a membrane protein with BADAN: Probing protein topology and local environment. Biophys. J..

[22] Cohen J.A., Highsmith S. (1997). An improved fit to Website osmotic pressure data. Biophys. J..

[23] Solioz M., Odermatt A. (1995). Copper and silver transport by CopB-ATPase in membrane vesicles of *Enterococcus hirae*. J. Biol. Chem..

[24] Jurkiewicz P., Sýkora J., Ol żyńska A., Humpolíčková J., Hof M. (2005). Solvent relaxation in phospholipid bilayers: Principles and recent applications. J. Fluoresc..

[25] Salustros N., Grønberg C., Abeyrathna N.S., Lyu P., Orädd F., Wang K., Andersson M., Meloni G., Gourdon P. (2022). Structural basis of ion uptake in copper-transporting P_1B_-type ATPases. Nat. Commun..

[26] Guo Z., Orädd F., Bågenholm V., Grønberg C., Ma J.F., Ott P., Wang Y., Andersson M., Pedersen P.A., Wang K. (2024). Diverse roles of the metal binding domains and transport mechanism of copper transporting P-type ATPases. Nat. Commun..

[27] Dyla M., Terry D.S., Kjaergaard M., Sørensen T.L.-M., Lauwring Andersen J., Andersen J.P., Rohde Knudsen C., Altman R.B., Nissen P., Blanchard S.C. (2017). Dynamics of P-type ATPase transport revealed by single-molecule FRET. Nature.

[28] Chawla U., Perera S.M.D.C., Fried S.D.E., Eitel A.R., Mertz B., Weerasinghe N., Pitman M.C., Struts A.V., Brown M.F. (2021). Activation of the G-Protein-Coupled Receptor Rhodopsin by Water. Angew. Chem. Int. Ed..

[29] Sohlenkamp C., Geiger O. (2016). Bacterial membrane lipids: Diversity in structures and pathways. FEMS Microbiol. Rev..

[30] Oursel D., Loutelier-Bourhis C., Orange N., Chevalier S., Norris V., Lange C.M. (2007). Lipid composition of membranes of *Escherichia coli* by liquid chromatography/tandem mass spectrometry using negative electrospray ionization. Rapid Commun. Mass. Spectrom..

[31] Michelangeli F., Orlowski S., Champeil P., Grimes E.A., East J.M., Lee A.G. (1990). Effects of phospholipids on binding of calcium to (Ca^2+^-Mg^2+^)-ATPase. Biochemistry.

[32] Michelangeli F., Grimes E.A., East J.M., Lee A.G. (1991). Effects of phospholipids on the function of (Ca^2+^-Mg^2+^)-ATPase. Biochemistry.

[33] Le Maire M., Champeil P., Møller J.V. (2000). Interaction of membrane proteins and lipids with solubilizing detergents. Biochim. Biophys. Acta Biomembr..

[34] Zimmer J., Doyle D.A. (2006). Phospholipid requirement and pH optimum for the in vitro enzymatic activity of the *E. coli* P-type ATPase ZntA. Biochim. Biophys. Acta Biomembr..

[35] Subramani S., Perdreau-Dahl H., Morth J.P. (2016). The magnesium transporter A is activated by cardiolipin and is highly sensitive to free magnesium in vitro. eLife.

[36] De Foresta B., Henao F., Champeil P. (1992). Kinetic characterization of the perturbation by dodecylmaltoside of sarcoplasmic reticulum Ca^2+^-ATPase. Eur. J. Biochem..

[37] De Foresta B., Henao F., Champeil P. (1994). Cancellation of the cooperativity of Ca^2+^ binding to sarcoplasmic reticulum Ca^2+^-ATPase by the non-ionic detergent dodecylmaltoside. Eur. J. Biochem..

[38] Vilsen B., Andersen J.P. (1987). Effect of phospholipid, detergent and protein-protein interaction on stability and phosphoenzyme isomerization of soluble sarcoplasmic reticulum Ca-ATPase. Eur. J. Biochem..

[39] Danielczak B., Keller S. (2018). Collisional lipid exchange among DIBMA-encapsulated nanodiscs (DIBMALPs). Eur. Polym. J..

[40] Hoffmann M., Eisermann J., Schöffmann F.A., Das M., Vargas C., Keller S., Hinderberger D. (2021). Influence of different polymer belts on lipid properties in nanodiscs characterized by CW EPR spectroscopy. Biochim. Biophys. Acta Biomembr..

[41] Picard M., Dahmane T., Garrigos M., Gauron C., Giusti F., le Maire M., Popot J.-L., Champeil P. (2006). Protective and inhibitory effects of various types of amphipols on the Ca^2+^-ATPase from sarcoplasmic reticulum: A comparative study. Biochemistry.

[42] Sun C., Benlekbir S., Venkatakrishnan P., Wang Y., Hong S., Hosler J., Tajkhorshid E., Rubinstein J.L., Gennis R.B. (2018). Structure of the alternative complex III in a supercomplex with cytochrome oxidase. Nature.

[43] Marsh D. (1996). Lateral pressure in membranes. Biochim. Biophys. Acta Rev. Biomembr..

[44] Oluwole A.O., Danielczak B., Meister A., Babalola J.O., Vargas C., Keller S. (2017). Solubilization of Membrane Proteins into Functional Lipid-Bilayer Nanodiscs Using a Diisobutylene/Maleic Acid Copolymer. Angew. Chem. Int. Ed..

[45] Carter S.G., Karl D.W. (1982). Inorganic phosphate assay with malachite green: An improvement and evaluation. J. Biochem. Biophys. Methods.

[46] Wieczorek H., Cioffi M., Klein U., Harvey W.R., Schweikl H., Wolfersberger M.G. (1990). Isolation of goblet cell apical membrane from tobacco hornworm midgut and purification of its vacuolar-type ATPase. Methods Enzymol..

[47] Gonzalez-Tello P., Camacho F., Blazquez G. (1994). Density and Viscosity of Concentrated Aqueous Solutions of Polyethylene Glycol. J. Chem. Eng. Data.

